# Gradient-Type Magnetoelectric Current Sensor with Strong Multisource Noise Suppression

**DOI:** 10.3390/s18020588

**Published:** 2018-02-14

**Authors:** Mingji Zhang, Siu Wing Or

**Affiliations:** 1 Department of Electrical Engineering, The Hong Kong Polytechnic University, Hung Hom, Kowloon, Hong Kong, China; mingji.zhang@connect.polyu.hk; 2 Hong Kong Branch of National Rail Transit Electrification and Automation Engineering Technology Research Center, Hong Kong, China

**Keywords:** current sensor, magnetic field gradient, magnetoelectric effect, multisource noise suppression

## Abstract

A novel gradient-type magnetoelectric (ME) current sensor operating in magnetic field gradient (MFG) detection and conversion mode is developed based on a pair of ME composites that have a back-to-back capacitor configuration under a baseline separation and a magnetic biasing in an electrically-shielded and mechanically-enclosed housing. The physics behind the current sensing process is the product effect of the current-induced MFG effect associated with vortex magnetic fields of current-carrying cables (i.e., MFG detection) and the MFG-induced ME effect in the ME composite pair (i.e., MFG conversion). The sensor output voltage is directly obtained from the gradient ME voltage of the ME composite pair and is calibrated against cable current to give the current sensitivity. The current sensing performance of the sensor is evaluated, both theoretically and experimentally, under multisource noises of electric fields, magnetic fields, vibrations, and thermals. The sensor combines the merits of small nonlinearity in the current-induced MFG effect with those of high sensitivity and high common-mode noise rejection rate in the MFG-induced ME effect to achieve a high current sensitivity of 0.65–12.55 mV/A in the frequency range of 10 Hz–170 kHz, a small input-output nonlinearity of <500 ppm, a small thermal drift of <0.2%/℃ in the current range of 0–20 A, and a high common-mode noise rejection rate of 17–28 dB from multisource noises.

## 1. Introduction

Current sensors are of great importance in electrical condition monitoring for the purposes of planning for effective energy usage and fault prediction in modern electrical systems [1,2,3,4,5,6,7]. They are usually mounted on cables to sense cable currents and to produce electrical signals proportional to the cable currents. A major challenge to current sensors is the increasing types and levels of noises from multiple sources (i.e., multisource noises) as a result of increasingly complicated application environments. The rapid development of smart and safe electrified cities worldwide, especially for the enabling of smart grids and e-mobilities, has imposed a great demand for high-performance current sensors with an improved common-mode noise rejection rate (*CMRR*) for electric field noise, magnetic field noise, vibration noise, thermal noise, and simultaneously possessing high sensitivity, small input-output nonlinearity, small thermal drift, passive (i.e., power-free) sensing, compact size, and low cost [2,3,6]. For example, a modern railway electrification system may require thousands and even millions of current sensors to form a sensor network in various operating environments involving high electric field noise induced by high-voltage (>1 kV) apparatuses, high magnetic field noise caused by heavy-current (>100 A) cables, high vibration noise raise from high running speeds (>100 km/h), and/or large temperature variation (~40 °C) under different circumstances [6,8,9].

State-of-art current sensors such as current transformers, Rogowski coils, fluxgate sensors, and Hall sensors sense cable currents by detecting the strength of magnetic fields in the vicinity of the cables [1,7,9,10,11,12,13,14,15,16,17,18,19,20,21]. Current transformers allow passive sensing with a high accuracy of >95% in the large current range of ~1 A–1 kA. However, the need of a large core size and a high winding ratio gives rise to a bulky and heavy device with a limited frequency range of <5 kHz and a low *CMRR* of <10 dB [7,13,14]. Rogowski coils feature a core-free passive-sensing design to achieve a very small nonlinearity of <24 ppm, a large current range up to 1 MA, and a wide frequency range up to 1 MHz at the expense of a small sensitivity of ~0.1 mV/A [11,12,22]. Fluxgate sensors demonstrate a high sensitivity of ~28 mV/A at the cost of a small current range of <20 A, a small frequency range of <10 kHz, and requiring auxiliary electronics [19,23]. Hall sensors have the distinct advantages of compact size of ~1 cm, but they require a highly stable DC current supply to excite the Hall effect and a high-quality signal conditioner to process the inherently weak Hall voltages and to compensate for the serious thermal drift [18,24]. 

Recently, ME current sensors have received considerable research and application attention for AC current sensing by detecting the magnetic field strength in the vicinity of current-carrying cables in accordance with Ampere’s law and by converting the detected magnetic field strength into voltage on the basis of the ME effect in a single ME composite [20,21,25]. This is because of their distinct features of compacted size (~10 mm) and easy installation in comparison to current transformers and Rogowski coils; their large sensitivity (up to 0.5 V/A) in excess of 100 times over the Hall sensors; the fact they are free of external power supplies, signal conditioners, and/or other auxiliary means as normally required in the fluxgate sensors; and the added merits of small input-output nonlinearity (<0.5%) and small thermal drift (<1%/°C) [25,26,27,28,29]. However, it is found that these strength-type ME current sensors are subject, simultaneously, to electric field, magnetic field, and vibration noise sources when installed in a multisource noise environment (i.e., an e-mobility system). In this case, the ME voltage in the output will be submerged in voltage noises induced by multiple noise sources, and hence make the measurement of the current inaccurate, even impossible. Fortunately, our previous study has demonstrated the distinct performances of the high common-mode noise rejection rate and high sensitivity for the detection of MFG [30]. It shed light upon the sensing of the current by MFG detection and conversion based on the product effect of the current-induced MFG effect and the MFG-induced ME effect in the ME composite pair, respectively, to give a high multisource *CMRR* and a high measurement sensitivity. 

In this paper, we theoretically and experimentally report the realization of a novel gradient-type ME current sensor with gained merits of high sensitivity and high common-mode multisource noises rejection rate from current-induced MFG effect and the MFG-induced ME effect. An analytical model is derived to disclose the working principles of the sensor. Multisource noises coupling mechanism (i.e., magnetic fields noises, electric field noises, vibration noises, and thermal noises) are investigated and semi-empirically formulated. The performances of a sensor porotype are systematically characterized to achieve a high sensitivity of 0.65–12.55 mV/A in the frequency range of 10–170 kHz, a strong multisource *CMRR* of 17–28 dB, a small input-output nonlinearity of <500 ppm, and a small thermal drift of <0.2%/℃ within the measurement range of 0–20 A. 

## 2. Configuration, Structure, and Prototype

**Figure 1:**
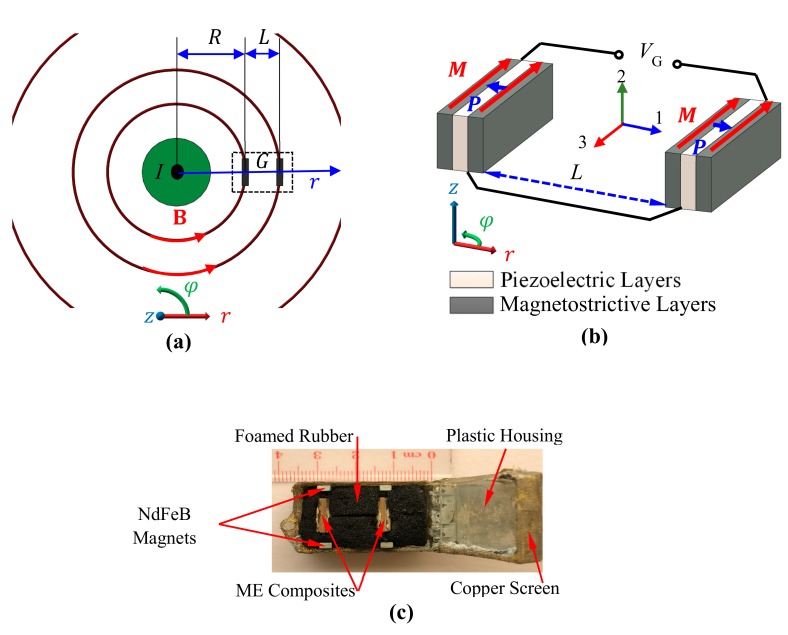
The novel gradient-type ME current sensor based on the product effect of current-induced MFG effect and the MFG-induced ME effect: (**a**) top-view of current sensor assembly configuration and magnetic fields (**B**) and its gradient (G) generated in the vicinity of a current (*I*)-carrying cable (**b**) structure of ME composites pair in the sensor, in which *M* denotes the magnetization direction of the magnetostrictive layers and *P* indicates the polarization direction of the piezoelectric layer; (**c**) packaged prototype.

Figure 1 shows the assembly configuration, structure, and prototype of the proposed gradient-type ME current sensor. The sensor consists of a pair of plate-shaped Terfenol-D/PZT/Terfenol-D tri-layer ME composites separated with a baseline (*L* = 16 mm) in the radial (*r*-) direction, biased by two pair of NdFeB magnets ($\bar{B_{\mathrm{bias}}}=42\;\mathrm{mT}$) tangentially to the circumferential (φ-) direction, and surrounded by foamed rubber in a plastic box with copper screen shielding (see Figure 1a,b). (0.05 mm wire diameter and 0.25 mm^2^ hole area). The sensor is symmetrically mounted and loosely contacted with the foamed rubber in the plastic box as in Figure 1c. The usage of copper screen is to minimize the influence of ambient electric field noises based on the conductive shielding effect, and to reduce the magnetic field attenuation due to eddy current effect by reducing effective eddy current vortexes. To enable the MFG-induced ME effect for current measurement, the two ME composites are prepared by bonding a layer of PZT (Pb(Zr, Ti)O_3_, Ceram-Tec P8) piezoelectric ceramic plate of 12 mm length, 6 mm width, and 1 mm thickness between two layers of [112]-textured Terfenol-D (Tb_0.3_Dy_0.7_Fe_1.92_, Baotou Rare Earth, Baotou, China) magnetostrictive alloy plates of the same dimensions, by cutting the ME composite along its length direction (3-) using dicing saw (DAD 321, Giorgio Technology, Mesa, AZ, USA) with lowest blade feeding speed of 0.2 mm/s, blade depth of 3 mm, and by separating the obtained ME composites with dimensions of 12 mm long, 3 mm wide, and 1 mm thick. The bias magnets are made of NdFeB (N55M, V-magnet, Shanghai, China) with dimensions of 6 mm long, 3 mm wide, and 2 mm thick. As in Figure 1b, the magnetization (*M*) direction of the Terfenol-D plates and the polarization (*P*) direction of the PZT plate are oriented in their length (3-) and thickness (1-) directions, respectively. The negative electrode surfaces of the PZT plate in the two ME composites are electrically connected together to form a back-to-back capacitor configuration, while the positive electrode surfaces of the first and second PZT plates are connected to the signal core and ground shield of the coaxial cable with BNC termination, respectively. Therefore, the output voltage is directly obtained from the ME composites pair, and is directly calibrated against current amplitude to give the current sensitivity characterized by a unit output voltage per unit ampere in the cable. 

## 3. Working Principle

### 3.1. Current Sensing Based on MFG Detection and Conversion

The working principle of the sensor can be described by detecting the current (I)-induced MFG (G) and converting the detected G into voltage ($V_{G}$) (i.e., MFG detection and conversion) in accordance with the product effect of the current-induced MFG effect and the MFG-induced ME effect. As shown in Figure 1a, the magnetic field strength *B* at the position *r* around a current-carrying cable is governed by:

(1) $$B=\frac{\mu_{0}I}{2\pi r_{\;}},$$

whose gradient (G) is defined as the spatial differential of B in the radial (-*r*) direction as:

(2) $$G=\frac{dB}{dr}=\frac{d}{dr}\left(\frac{\mu_{0}I}{2\pi r_{\;}}\right).$$

When an ME gradient sensor with gradient-ME voltage coefficient ($\alpha_{G}$) is assembled at certain position *R* (Figure 1a), the current-induced gradient-ME voltage ($V_{G}$) reads:

(3) $$V_{G}=\alpha_{G}G.$$

Defining the sensitivity of the gradient-type ME current sensor ($S_{I}$) as a unit voltage output per unit current, $S_{I}$ can be derived from Equations (1)–(3) as:

(4) $$S_{I}=\frac{dV_{G}}{dI}=\frac{dV_{G}}{dG}\frac{dG}{dI}=\alpha_{G}\frac{d}{dI}\left(\frac{dB}{dr}\right).$$

The right-hand side in Equation (4) demonstrates the products mechanism of current-induced MFG effect and the MFG-induced ME effect in the proposed current sensor, in which the MFG-induced ME effect is quantified by $\alpha_{G}$ and the current-induced MFG is represented by the remaining [30]. The middle parts in Equations (4) also provide an alternative interpretation on the working principle of the sensor as an MFG-mediated ME current sensor.

Practically, the spatial differential of B in Equation (4) is obtained by spatially differencing $B_{A}$ and $B_{B}$ over the baseline *L* in radial (*r*-) direction as:

(5) $$\frac{dB}{dr}=\frac{B_{A}-B_{B}}{L}=\frac{\mu_{0}}{2\pi R_{\;}\left(R+L\right)}I.$$

By substituting Equation (5) into Equation (4), the designed $S_{I}$ of the sensor as function of assembly parameters (R and L) turns out to be

(6) $$S_{I}=\frac{\alpha_{G}\mu_{0}}{2\pi R_{\;}\left(R+L\right)}.$$

### 3.2. Coupling and Suppression of Multsource Noises

The output voltage $V_{G}$ from the ME composites pair can be also theoretically expressed as in Equation (7) because of the back-to-back capacitor configuration in Figure 1b.

(7) $$V_{G}=V_{M,A}-V_{M,B},$$

in which $V_{M,A}$ and $V_{M,B}$ are superposition of ME voltages in each ME composites and total voltage noises (${\tilde{v}}_{M,A}$ and ${\tilde{v}}_{M,B}$) as in Equation (8).

(8) $$V_{M,i}=\alpha_{V,i}B_{i}+{\tilde{v}}_{M,i}.(i=A,\;B)$$

in which $\alpha_{V,i}$ in Equation (8) is the conventional ME voltage coefficients. 

**Figure 2:**
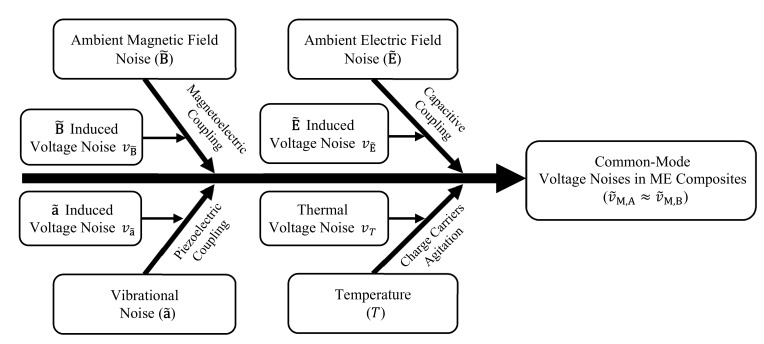
Ishikawa diagram of multisource noise coupling mechanism in the proposed gradient-type ME current sensor.

Figure 2 shows the multisource noise coupling mechanism in the sensor using Ishikawa diagram. In Equation (8), ${\tilde{v}}_{M,A}$ and ${\tilde{v}}_{M,B}$ are contributed by magnetic fields noise ($\tilde{B}$)-induced voltage noise ($v_{\tilde{B}}$), electric field noise ($\tilde{E}$)-induced voltage noise ($v_{\tilde{E}}$), vibration noise ($\tilde{a}$)-induced voltage noise ($v_{\tilde{a}}$), and thermal voltage noise ($v_{T}$) as:

(9) $${\tilde{v}}_{M,A}\approx {\tilde{v}}_{M,B}=v_{\tilde{B}}+v_{\tilde{E}}+v_{\tilde{a}}+v_{T},$$

in which $\tilde{B}$ is coupled into $v_{\tilde{B}}$ following the conventional ME effect as:

(10) $$v_{\tilde{B}}=\alpha_{V}\tilde{\left|B\right|.}$$

In Equation (9), $\tilde{E}$ is coupled into $v_{\tilde{E}}$ by means of capacitive coupling. For open-circuit condition, $v_{\tilde{E}}$ can be expressed as:

(11) $$v_{\tilde{E}}=2\pi fR_{L}\overset{\;}{\underset{\Gamma }{\int }}\left(\epsilon_{0}\epsilon_{r}\tilde{E}\right)\cdot ndS,$$

in which $R_{L}\geq 1\;M\Omega$ is the input resistance of external circuit, f the frequency, $\epsilon_{0}$ the vaccum permitivity, $\epsilon_{r}$ the relative permittivity matrix, n the normal direction vector of the electrode, Γ the surface geometry of a positive electrode, and d*S* the surface element of a positive electrode.

In Equation (9), $v_{\tilde{a}}$ is mainly contributed by acceleration *(*$\tilde{a}$*)*-induced piezoelectric voltage. The amplitude of $v_{\tilde{a}}$ is determined by the shape and assembly conditions of the test platform; however, a semi-empirical form of $v_{\tilde{a}}$ under open-circuit condition can be expressed as function of $\tilde{a}$ given in Equation (12).

(12) $$v_{\tilde{a}}=2\pi fR_{L}\rho d\overset{\;}{\underset{\Gamma }{\int }}\left(e_{\mathrm{PE}}^{E}{\left(C^{E}\right)}^{-1}\tilde{a}\right)\cdot ndS$$

in which ρ is the average density of an ME composite, d the thickness of piezoelectric plate, $e_{\mathrm{PE}}^{E}$ the piezoelectric the coupling matrix in stress-charge form under a constant electric field, and $C^{E}$ the fourth-order elastic matrix of the piezoelectric plate under constant static electric field [30].

In Equation (9), $v_{T}$ is temperature (*T*) and frequency (*f*)-dependent noises (thermal noises) contributed by dielectric loss noise and 1/*f* noise, which are written as shown by the first and second term in Equation (13), respectively.

(13) $$v_{T}=\sqrt{\frac{4k_{B}T\tan\left(\delta \right)\cdot f}{2\pi fC}}+\frac{1}{2\pi fC}\sqrt{\frac{4k_{B}T\Delta f}{R_{L}}},$$

in which δ, k, and T are dielectric loss factor, Boltzmann’s constant, and temperature, respectively. Δf and C are the equivalent frequency band width of measurement equipment and capacitor of the ME composite, respectively [31]. 

When the gradient-type ME current sensor is working in multisource noises-interfered environment to sense I in a cable, voltage noises in each ME composite are essentially common-mode noises (${\tilde{v}}_{M,A}\approx {\tilde{v}}_{M,B}$). Therefore, the real output voltage from the sensor ($V_{G}$) can be derived by combining Equations (6)–(9) in form of the superposition of current signal part and noise part (${\tilde{v}}_{G}$):

(14) $$V_{G}=\frac{\alpha_{G}\mu_{0}}{2\pi R_{\;}\left(R+L\right)}I+{\tilde{v}}_{G},$$

in which ${\tilde{v}}_{G}$ is the suppressed total voltage noises from the current sensor written as:

(15) $${\tilde{v}}_{G}={\tilde{v}}_{M,A}-{\tilde{v}}_{M,B}\approx 0.$$

To evaluate the noise suppression performance, the *CMRR* of gradient-type ME current sensor is defined as the ratio of the powers in ${\tilde{v}}_{G}$ over that of ${\tilde{v}}_{M,i}$ in unit of positive decibel as:

(16) $$CMRR=-20\log_{10}\left(\frac{{\tilde{v}}_{G}}{{\tilde{v}}_{M,i}}\right)\;\mathrm{dB}.$$

## 4. Performance Evaluation, Results, and Discussion

### 4.1. Intrincsic Performance

#### 4.1.1. Evaluation of Voltage Coefficient of MFG-Induced ME Effect

**Figure 3:**
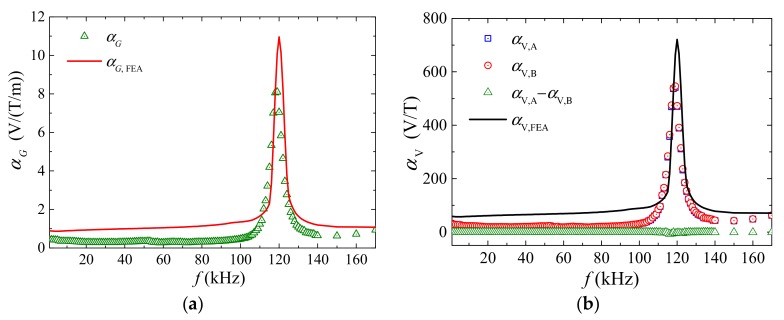
Frequency (*f*) dependence of: (**a**) measured gradient-ME voltage coefficient $\alpha_{G\;}$ and its calculated results from FEM ($\alpha_{G,\;\mathrm{FEA}}$); (**b**) measured ME voltage coefficients of the two ME composites ($\alpha_{V,A\;}$ and $\alpha_{V,B\;}$) and their difference ($\alpha_{V,A}-\alpha_{V,B}$), together with that of the ME voltage coefficient calculated from FEA ($\alpha_{\mathrm{FEA}}$).

Figure 3a plots the frequency (*f*) dependence of the measured and calculated gradient-ME voltage coefficient ($\alpha_{G\;}$ and $\alpha_{G,\;\mathrm{FEA}}$) of the sensor using the methods in [30]. The $\alpha_{G\;}$ and $\alpha_{G,\;\mathrm{FEA}}$ spectra are quantitatively similar to the $\alpha_{V\;}$ spectra in Figure 3b, suggesting the determination of $\alpha_{G\;}$ by $\alpha_{V,A\;}$ and $\alpha_{V,B\;}$ described by Equation (8). Non-resonance $\alpha_{G\;}$ of 0.34 V/T/m is achieved over a broad frequency range of 1 Hz–80 kHz, while the highest $\alpha_{G\;}$ of 8.6 V/(T/m) is obtained at resonance frequency, $f_{r}$ of 120 kHz. 

Figure 3b plots the frequency (*f*) dependence of the measured ME voltage coefficients of the two ME composites ($\alpha_{V,A\;}$ and $\alpha_{V,B\;}$) and their difference ($\alpha_{V,A}-\alpha_{V,B\;}$) for comparison with that of the ME voltage coefficient calculated from FEA ($\alpha_{\mathrm{FEA}}$) following the modeling methods in [30]. It is seen from Figure 3b that the $\alpha_{V,A\;}$ and $\alpha_{V,B\;}$ spectra have a difference ($\alpha_{V,A}-\alpha_{V,B}$) that fluctuates about zero in the frequency range of 1 Hz–170 kHz with relative error ($2\left(\alpha_{V,A}-\alpha_{V,B}\right)$/$\;(\alpha_{V,A}+\alpha_{V,B}$)) < 0.5%, which is one-sixth of that in [30]. This excellent similarity in the two ME composites is enabled by the improved manufacturing method described in Section 2, and well supports the condition of $\alpha_{V,A}≅\alpha_{V,B}$ of the ME composites pair in the current sensor. Both $\alpha_{V,A\;}$ and $\;\alpha_{V,B\;}$ exhibit a resonance peak of ~541 V/T at the resonance frequency of 120 kHz, corresponding to a half longitudinal wavelength of 12 mm in the ME composites. Non-resonance $\alpha_{V,\;A\;}$ and $\;\alpha_{V,B\;}$ has an average value of 28 V/T from 1 Hz to 100 kHz.

#### 4.1.2. Calibration of Current Sensitivity

**Figure 4:**
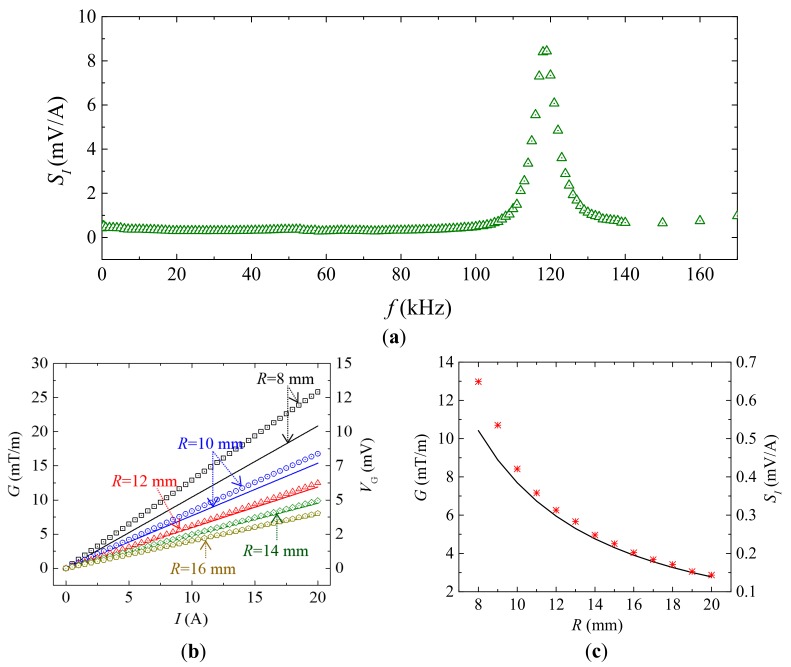
(**a**) Frequency (*f*) dependence of the measured current sensitivity ($S_{I}$) when *R* = 8 mm, (**b**) measured (symbols) and calculated (solid lines) G and $V_{G}$ various current amplitude at 50 Hz, and (**c**) measured (symbols) and calculated (solid lines) G and $S_{I}$ at various *R* of 8–20 mm at 50 Hz.

Figure 4a shows the frequency (*f*) dependence of the measured current sensitivity ($S_{I}$) when *R* = 8 mm. The $S_{I}$ spectrum was obtained by fixing the current sensor around a straight cable (AWG-10) in the *r*–φ plane as in Figure 1a, by producing an AC reference voltage of constant amplitude over the prescribed frequency range of 1 Hz–170 kHz using a lock-in amplifier (SRS^®^ SR865, Sunnyvale, CA, USA), by converting and amplifying the AC reference voltage into the corresponding AC current using a current supply amplifier (AE Techron^®^ 7548, Elkhart, IN, USA), by driving the cable with the AC current of 0–20 A in steps of 0.5 A, by measuring $V_{G}$ using the lock-in amplifier, and by calculating the slope of $V_{G}–I$ curves at each frequency. The AC current was monitored and assured using a current probe (HIOKI^®^ 9273, Nagano, Japan) and a signal conditioner (HIOKI^®^ 3271) connected to the current feedback input of the lock-in amplifier. An average of non-resonance $S_{I}$ of 0.62 mV/A is achieved over a broad frequency range of 1 Hz–80 kHz, while high resonance $S_{I}$ of 8.4 mV/A is obtained at $f_{r}$ of 120 kHz. The similarity trends in Figure 3a and Figure 4a implicit a linear relationship between current-induced MFG effect and the MFG-induced ME effect as described by Equations (4) and (6). 

Figure 4b shows the measured (symbols) and calculated (solid lines)$\;V_{G}$ and G at various current amplitude of 0–20 A at 50 Hz when *R* = 8, 10, 12, 14, and 16 mm, respectively. Measured G in Figure 4b was obtained by dividing measured $V_{G}$ over measured $\alpha_{G}$ in Figure 3a, while calculated G and $V_{G}$ were obtained using Equations (2) and (3), respectively. The difference between measured and calculated results, when *R* < 10 mm, can be explained by the flux distraction effects in the Terfenol-D plates when the ME composites is close to the cable. However, the straight curves in Figure 4b suggest that flux distraction effects will not affect the accuracy and input-output non-linearity of the current sensor. A very small input-output nonlinearity is evaluated to be <500 ppm in the current range of 0–20 A. 

Figure 4c shows the measured (symbols) and calculated (solid lines) G and $S_{I}$ at various *R*. G was obtained using the same method as Figure 4b, while $S_{I}$ was obtained by evaluating the slopes of $V_{G}–I$ curves in Figure 4b. Figure 4c indicates that a maximum $S_{I}$ of 0.65 mV/A is achieved at *R* = 8 mm. This result suggests an optimal assembly configuration that fixing the current sensor closer to the cable center will results in higher sensitivity. The obtained sensitivity is six-times higher than that of general Hall elements current sensors and Rogowski coils.

#### 4.1.3. Evaluation of Equivlent Current Noise Density

**Figure 5:**
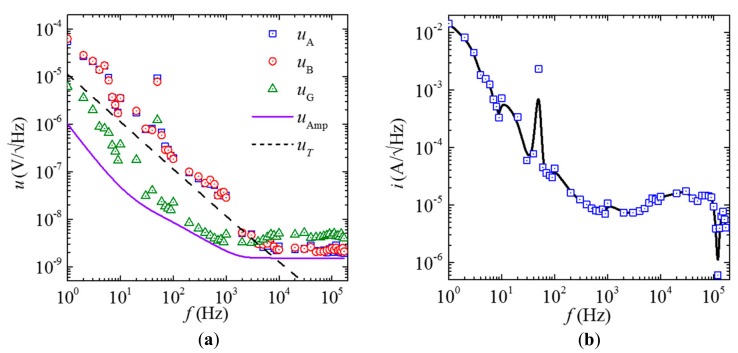
(**a**) Measured voltage noise density spectra of each ME composites ($u_{A}$ and $u_{B}$), the current sensor ($u_{G}$), and the lock-in amplifier noise floor ($u_{\mathrm{Amp}}$), together with the calculated thermal noise density ($u_{T}$) spectra. (**b**) Measured (symbols) and fitted (solid lines) equivalent current noise density (*i*) spectra in a magnetically-unshielded laboratory environment at 20 °C without intentional interference.

Figure 5a shows the measured voltage noise density spectra of each ME composites ($u_{A}$ and $u_{B}$), the current sensor ($u_{G}$), and the lock-in amplifier noise floor ($u_{\mathrm{Amp}}$), together with the calculated thermal noise density ($u_{T}$) spectra in a magnetically-unshielded laboratory environment at 20 °C without intentional interference. The spectra were obtained using the same method as in [30]. Since the effect of the ambient electric field noise is minimized by the copper screen shielding, the $u_{A}$, $u_{B}$, and $u_{G}$ spectra in Figure 5 are dominated by the thermal noises from 1 Hz to 3 kHz. In the frequency range of 20–150 Hz, the power-frequency noise arising from the magnetically-unshielded laboratory environment has an obviously added effect on $u_{G}$. In the further elevated frequency range of 3–170 kHz, the noise associated with measurements circuit is active since $u_{A}$, $u_{B}$, and $u_{G}$ all fluctuate over constant values. Beyond 3 kHz, $u_{G}$ becomes double of $u_{A}$ and $u_{B}$, which can be explained by the phase-lag between the voltage noises (${\tilde{v}}_{M,A}$ and ${\tilde{v}}_{M,B}$) in the two ME composites at high *f* because the voltage noises have random phases at high *f* (i.e., f  > 3 kHz). 

Figure 5b shows the intrinsic equivalent current noise density (*i*) spectrum in a magnetically-unshielded laboratory environment at 20 °C without intentional interference. The *i* spectrum was evaluated using measured $u_{G}$ in Figure 5a devided by measured $S_{I}$ in Figure 4a at each frequency in a like manner to the methods in [31]. It is found that the proposed gradient-type ME current sensor has intrinsic equivalent current noise density of 0.6 μA/$\sqrt{\mathrm{Hz}}$–14.3 mA/$\sqrt{\mathrm{Hz}}$ in the frequency range of 1 Hz–170 kHz.

### 4.2. Extrincsic Performance

#### 4.2.1. Evaluation of Thermal Stability

**Figure 6:**
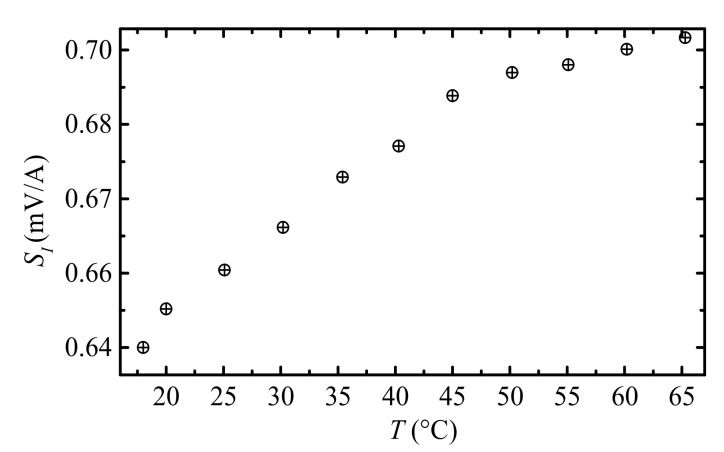
Experiment results of thermal stability: temperature (*T*) dependence of current measurement sensitivity ($S_{I}$).

Figure 6 shows the measured temperature (*T*) dependence of current measurement sensitivity ($S_{I}$). The $S_{I}$–T curve characterized in a modified non-magnetic turbo convection oven (Fangchu^®^, Guangzhou, China), with two via holes on the top and bottom covers to enable the straight current carrying cable pass through the oven. By controlling the temperature slowly increase from 18 °C to 65 °C with step of 5 °C and 10 min holding time to ensure thermal equilibrium state of the system, the $S_{I}$ at each temperature is obtained using the method described in Section 4.1.2. We see from Figure 6 that an increasing trend of $S_{I}$ as function of *T* can be found within 18–65 °C. The $S_{I}$. approximately linearly increases in the temperature range of 18–65 °C, while it decreases when *T* > 50 °C. The curve is in agreement with previous studies on thermal stability of ME composites, and may explained by increasing trend in temperature-dependent piezoelectric charge coefficients of PZT-8 plates and the soften effects of epoxy hardener at higher temperatures [28]. In detail, at low temperature range (18–50 °C), the rapid increasing trend of $S_{I}$ can be attributed to piezoelectric charge coefficient of PZT-8 plates. However, when temperature continues to increase (*T* > 50 °C), the epoxy becomes soft and consequently weakens the mechanical coupling between Terfenol-D plates and PZT-8 plates, resulting in the slow increase of $S_{I}$ as function of *T*. An overall relative sensitivity drift of <0.2%/°C is achieved in the temperature range of 18–65 °C. 

#### 4.2.2. Evaluation of Magnetic Fields Noise

**Figure 7:**
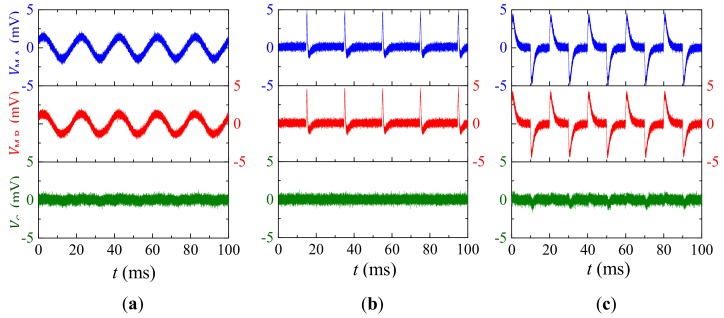
Experimental results of ambient magnetic field noises ($\tilde{B}$)-induced voltage noises in $V_{M,A}$, $V_{M,B}$, and $V_{G}$ when $\tilde{B}$ is created by (**a**) sine wave, (**b**) pulse, and (**c**) square wave current excitation, respectively.

Figure 7 shows the experiment waveforms of $\tilde{B}$-induced $v_{\tilde{B}}$ in $V_{M,A}$ and $V_{M,B}$, and ${\tilde{v}}_{G}$ in $V_{G}$ when the $\tilde{B}$ is artificially created in sine, pulse, and square waveforms, respectively. The waveforms in Figure 7 are experimentally obtained by settling a Helmholtz coil approximately 0.2 m away from the current sensor, by exciting the Helmholtz coil with a constant current supply amplifier (AE Techron^®^ 7548, Elkhart, IN, USA) connected to an arbitrary waveform generator (Agilent^®^ 33210A, Santa Clara, CA, USA), by monitoring the current in the coil with a current probe (HIOKI^®^ 9273, Nagano, Japan) and it signal conditioner (HIOKI^®^ 3271, Nagano, Japan), and by recording $V_{M,\;A}$, $V_{M,\;B}$, and $V_{G}$ using an oscilloscope (Tektronix^®^ MSO2014, OR, USA). Results in Figure 7a indicate that the 50 Hz component of ${\tilde{v}}_{M,A}$ and ${\tilde{v}}_{M,B}$ are of same amplitude of 1.5 mV, while the 50 Hz component of ${\tilde{v}}_{G}$ in $V_{G}$ is 0.2 mV. The ${\tilde{v}}_{G}$ in $V_{G}$ is evaluated to be 7 times smaller than $v_{\tilde{B}}$ in $V_{M,\;A}$ and $V_{M,\;B}$, corresponding to a *CMRR* of 17 dB in a $\tilde{B}$ interfered environment arising from ambient power cables. Results in Figure 7b are obtained by exciting the Helmholtz coil using pulse width-modulated current signal with pulse width of 10 μs, rising edge of 40 ns, and period of 20 ms in analogous to possible discharge current-induced $\tilde{B}$. Waveforms in Figure 7b indicate that the peak value of ${\tilde{v}}_{M,A}$ and ${\tilde{v}}_{M,B}$ are of same amplitude of 5 mV, while the suppressed peak value of ${\tilde{v}}_{G}$ in $V_{G}$ waveforms is less than 0.2 mV. The ${\tilde{v}}_{G}$ in $V_{G}$ is evaluated to be 12 times smaller than $v_{\tilde{B}}$ in $V_{M,\;A}$ and $V_{M,\;B}$, corresponding to a *CMRR* of 28 dB. The larger *CMRR* in this case can be explained by high frequency component in the pulse signal and high $\alpha_{G}$ in Figure 3a. The zero-crossing waveforms in Figure 7b can be explained by eddy current effects and magnetostrictive effects in Terfenol-D plates. Results in Figure 7c are obtained by exciting the Helmholtz coil using square wave current signal with period of 20 ms and rising edge of 0.1 ms in analogous to possible $\tilde{B}$ induced by pulse-width modulated actuator in the electrical system. Results in Figure 7c have similar trends to those in Figure 7b, except for the larger transition time at the edge of square wave, which can be explained by the parasitic capacitance, eddy current effects, and magnetostrictive effects in Terfenol-D plates. The ${\tilde{v}}_{G}$ in $V_{G}$ is evaluated to be 12 times smaller than $v_{\tilde{B}}$ in $V_{M,\;A}$ and $V_{M,\;B}$, corresponding to a *CMRR* of 21 dB. 

To summarize, results in Figure 7 obviously suggest that $\tilde{B}$-induced $v_{\tilde{B}}$ can be suppressed up to 25 times, corresponding to a *CMRR* up to 28 dB in the present gradient-type ME current sensor. However, it is interesting to find that the high frequency noise components of ${\tilde{v}}_{G}$ are almost 1.4 times that of $v_{\tilde{B}}$, because high frequency thermal noises with random phase lag between $V_{M,\;A}$ and $V_{M,\;B}$ result in doubled ${\tilde{v}}_{G}$.

#### 4.2.3. Evaluation of Electric Field Noise

**Figure 8:**
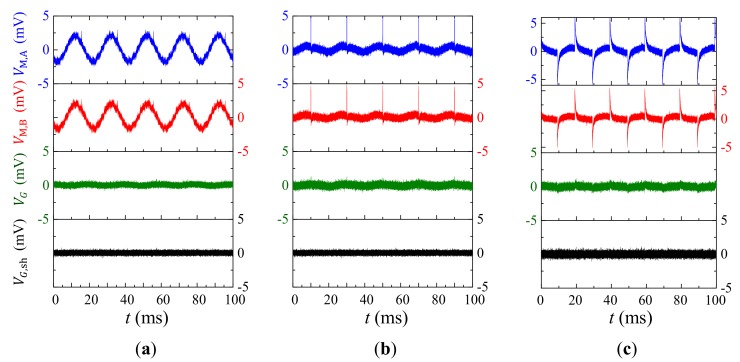
Experimental results of ambient electric field noises: ($\tilde{E}$)-induced voltage noises in $V_{M,A}$, $V_{M,B}$, and $V_{G}$ when $\tilde{E}$ is created by exciting (**a**) sine wave, (**b**) pulse width modulated, and (**c**) square voltage signals on two copper plates, respectively. (The peak-value of $\left|\tilde{E}\right|$ is approximately 1000 V/m for all cases).

Figure 8 shows the experiment waveforms of $\tilde{E}$-induced $v_{\tilde{E}}$ in $V_{M,A}$ and $V_{M,B}$, and ${\tilde{v}}_{G}$ in $V_{G}$ when the $\tilde{E}$ is artificially created in sine, pulse, and square waveforms, respectively. Experiments are conducted by assembling the sensor in the middle of two home-made copper plates with separating distance of 50 mm, by applying voltage excitation to the two copper plates by voltage amplifier (AE Techron^®^ 7548, Elkhart, IN, USA) connected to an arbitrary waveform generator (Agilent^®^ 33210A, Santa Clara, CA, USA), and by recording $V_{M,\;A}$, $V_{M,\;B}$,and $V_{G}$ using an oscilloscope (Tektronix^®^ MSO2014, Beaverton, OR, USA). The two coppers plates both have width, length, and thickness of 100 mm, 100 mm, and 1 mm, respectively. Figure 8a shows the waveforms of $V_{M,\;A}$, $V_{M,\;B}$, and $V_{G}$ when the excitation voltage is sine wave with amplitude of 50 V ($\left|\tilde{E}\right|=1000\;V/m$). Figure 8a suggest that 50 Hz components of $v_{\tilde{E}}$ in $V_{M,\;A}$ and $V_{M,\;B}$ amplitudes are approximately 2.1 mV, while the 50 Hz component of ${\tilde{v}}_{G}$ in $V_{G}$ is 0.18 mV. The ${\tilde{v}}_{G}$ in $V_{G}$ is evaluated to be 11 times smaller than $v_{\tilde{E}}$ in $V_{M,\;A}$ and $V_{M,\;B}$, and the *CMRR* is evaluated to be 21 dB without the copper screen shielding. Figure 8b shows the $\tilde{E}$-induced waveforms of $V_{M,\;A}$, $V_{M,\;B}$, and $V_{G}$ when the copper plates are driven by pulse width-modulated voltage signal with pulse width of 10 μs, rising edge of 40 ns, and period of 20 ms in analogous to possible, voltage-induced $\tilde{E}$ raising from the ON/OFF action of the electrical system. Figure 8b suggests that the peak values of $v_{\tilde{E}}$ in $V_{M,\;A}$ and $V_{M,\;B}$ are approximately 5 mV, while peak values of ${\tilde{v}}_{G}$ in $V_{G}$ are 0.7 mV. The ${\tilde{v}}_{G}$ in $V_{G}$ is evaluated to be 7 times smaller than $v_{\tilde{E}}$. in $V_{M,\;A}$ and $V_{M,\;B}$, and the *CMRR* is evaluated to be 17 dB without the copper screen shielding. Figure 8c shows the $\tilde{E}$-induced waveforms of $V_{M,\;A}$, $V_{M,\;B}$, and $V_{G}$ when the copper plates are driven by square voltage signal at 50 Hz. Figure 8c suggests that the peak values of $v_{\tilde{E}}$ in $V_{M,\;A}$ and $V_{M,\;B}$ are approximately 5.8 mV, while peak values of ${\tilde{v}}_{G}$ in $V_{G}$ are 0.8 mV. The ${\tilde{v}}_{G}$ in $V_{G}$ is evaluated to be 7.2 times smaller than $v_{\tilde{E}}$ in $V_{M,\;A}$ and $V_{M,\;B}$, and the *CMRR* is evaluated to be 17 dB without the copper screen shielding. 

Detail investigation on $V_{G}$ waveforms in Figure 8 indicates that the $\tilde{E}$-induced voltage noises are not completely suppressed, which may attribute to the unbalanced charge accumulation on the output electrodes of the sensor, as well as the sensor’s asymmetry assembly errors among the copper plates. However, by adding a grounded copper screen, $v_{\tilde{E}}$ in $V_{M,\;A}$ and $V_{M,\;B}$ are significantly reduced to give an extremely small ${\tilde{v}}_{G}$ in $V_{G}$ as shown in the bottom parts in Figure 8. For the grounded copper screen to shielded gradient-type ME current sensor, ${\tilde{v}}_{G}$ can be as 60 times smaller than that of $v_{\tilde{E}}$ in $V_{M,\;A}$ and $V_{M,\;B}$ in an un-shielded condition. 

#### 4.2.4. Evaluation of Vibration Noise

**Figure 9:**
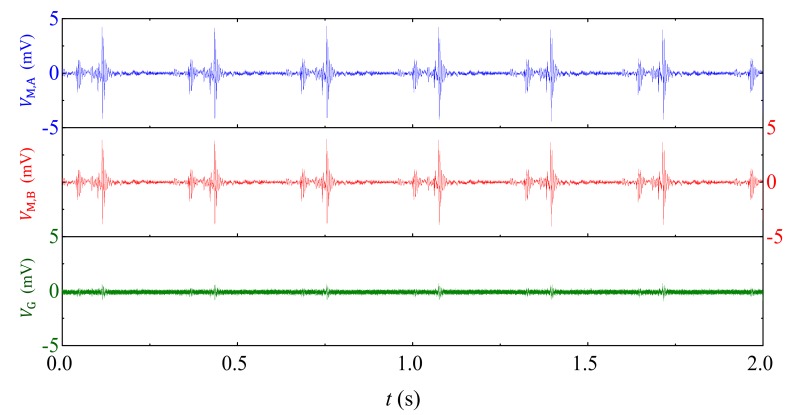
Experimental results of vibrational acceleration noises: ($\tilde{a}$)-induced voltage noises in $V_{M,A}$, $V_{M,B}$, and $V_{G}$.

Figure 9 shows the experiment waveforms of $\tilde{a}$-induced $v_{\tilde{a}}$ in $V_{M,A}$ and $V_{M,B}$, and ${\tilde{v}}_{G}$ in $V_{G}$. The experiment is conducted by fixing the sensor on Polytetrafluoroethylene (PTFE)-made holder clamped by non-magnetic clamps on a micro testers (Instron^®^ 5948, Norwood, MA, USA); by controlling the head to move in triangular wave form with period of 0.64 s and amplitude of 5 mm to a square wave form of velocity with amplitude of 15.6 mm/s and period of 0.64 s, and pulsed acceleration with peak value of 18.35 $m/s^{2}$ with period of 0.32 s; and by recording $V_{M,\;A}$, $V_{M,\;B}$, and $V_{G}$ using an oscilloscope (Tektronix^®^ MSO2014, Beaverton, OR, USA). Results in Figure 9 indicate that a pulsed $\tilde{a}$ with $\left|\tilde{a}\right|=18.35\;m/s^{2}$ will raise $v_{\tilde{a}}$ in $V_{M,\;A}$ and $V_{M,\;B}$ up to 4.3 mV in conventional strength-type ME current sensor; however, $v_{a}$ in $V_{G}$ is more than 8 times reduced to 0.49 mV based on the MFG-induced ME effect in the gradient-type current sensor. The *CMRR* is evaluated to be 18 dB in the present case. A little difference between waveforms of $V_{M,\;A}$ and $V_{M,\;B}$ can be found in Figure 9, which may be caused by difference in mode shape asymmetry of the holder. However, it is safe to say that the *CMRR* will be much higher in the moving train sets of HSR system, since larger platforms vibrate more evenly, resulting in smaller acceleration difference at each ME composite. 

## 5. Suppression of Multisource Noises

### 5.1. Experiment Setup

**Figure 10:**
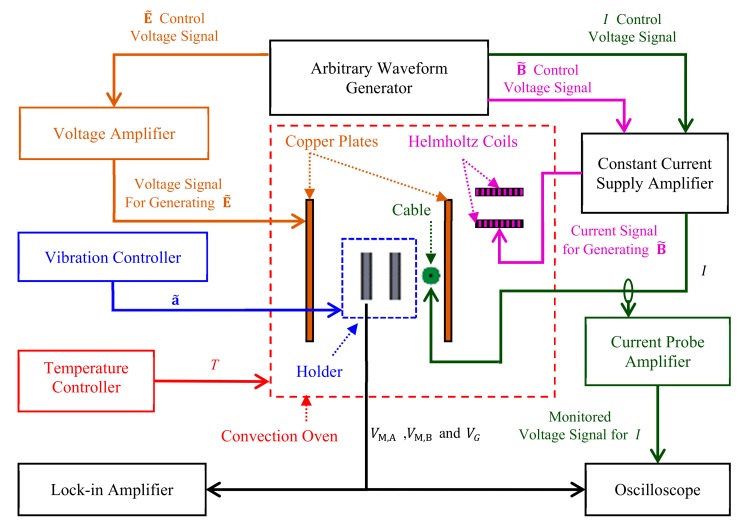
Schematics diagram of experiment setup for multisource noise suppression performance evaluation. Labels, lines, and arrows with colors of magenta, brown, blue, green, and red represent $\tilde{B}$-, $\tilde{E}$-, $\tilde{a}$-, I, and *T*-related equipments, respectively.

**Figure 11:**
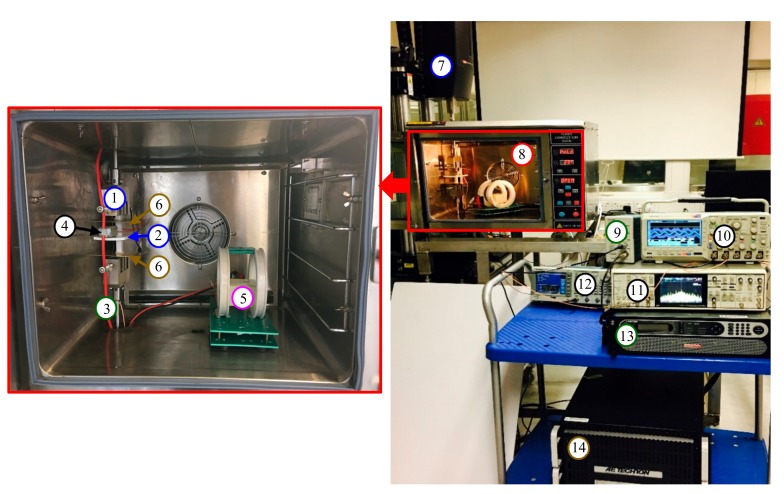
Experiment system for multisource noise suppression performance evaluation. Labels with colors of magenta, brown, blue, green, and red represent $\tilde{B}$-, $\tilde{E}$-, $\tilde{a}$-, I-, and *T*-related equipment, respectively. (1. Vibration head; 2. Holder; 3. Cable; 4. Sensor; 5. Helmholtz coils; 6. Copper plates; 7. Vibration controller; 8. Convection oven; 9. Current probe amplifier; 10. Oscilloscope; 11. Lock-in amplifier; 12. Arbitrary waveform generator; 13. Constant-current supply amplifier; 14. Constant-voltage amplifier).

Figure 10 shows the schematics diagram of the experiment setup for multisource noise suppression performance evaluation. The experiment system (Figure 11) integrated the experiment designs in Section 4.2 to evaluate the performance of the sensor under simultaneously the interference of $\tilde{B}$, $\tilde{E}$, $\tilde{a}$ under constant temperature (*T*). The experiment is conducted by simultaneously generating $\tilde{B}$ using a Helmholtz coil driven by pulse width-modulated current with pulse width of 10 μs, rising edge of 40 ns, and period of 20 ms as described in Section 4.2.2, by generating square wave $\tilde{E}$ with amplitude of 1000 V/m as described in Section 4.2.3, by generating pulse $\tilde{a}$ with peak value of 6 $m/s^{2}$ as described in Section 4.2.4, and by exciting the cable using controlled sinewave *I* with amplitude of 1 A at 50 Hz as described in Section 4.1.2 at temperature of 25 ℃ maintained by the turbo convention oven described in Section 4.2.1.

### 5.2. Results and Discussion

**Figure 12:**
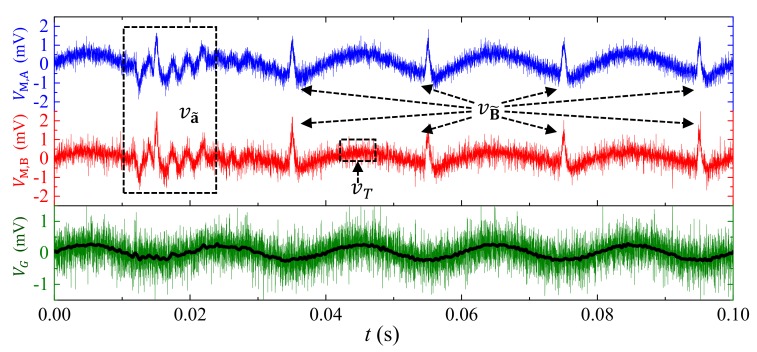
Experiment waveforms of $V_{M,A}$and $V_{M,B}$, and $V_{G}$, (green for raw data waveform, and black for 2-kHz low-pass filtered waveform) when measuring *I* in the cable under simultaneously interference of $\tilde{B}$, $\tilde{E}$, $\tilde{a}$, and thermal noises.

Figure 12 shows the experiment waveforms of $V_{M,A}$ and $V_{M,B}$, and $V_{G}$, when measuring *I* under simultaneously interference of $\tilde{B}$, $\tilde{E}$, $\mathrm{and}\;\tilde{a}$ using the integrated experiment system for multisource noise suppression performance evaluation described in Section 5.1. It can be seen from Figure 12 that the $v_{\tilde{B}},v_{\tilde{E}},v_{\tilde{a}}$ and $v_{T}$ in $V_{M,A}$ and $V_{M,B}$ have peak values of 1.5 mV, ~0 mV, 1.6 mV, and 0.7 mV, while the amplitude of 50 Hz sine waveform component in $V_{M,A}$ is 1 mV. The $v_{\tilde{E}}$ is too small to be observe because of the MFG-induced ME effect in the sensor and shielding effect of the copper screen. The signal to noise ratio in is evaluated to be 0.63 without applying gradient-type ME current sensor. When applied, the amplitude of 50 Hz sine waveform component in $V_{G}$ is evaluated to be 0.8 mV. We may find that the high frequency component $v_{T}$ is doubled because of random phase lag between $V_{M,\;A}$ and $V_{M,\;B}$; however, this can be solved with the help of a low-pass filter. With the help of a 2-kHz low-pass FFT filter, the obtained thermal noise in $V_{G}$ is less than 0.15 mV, and the obtained signal to noise ratio is evaluated to be 5.3 in the present case. During the experiment, it is interesting to find the average level of $V_{G}$ is much stable than that of $V_{M,A}$ and $V_{M,B}$. This can be explained by the low-frequency $v_{T}$ suppression enabled by MFG-induced ME effect in the sensor. To summarize, in the present experiment, the suppressions of $v_{\tilde{B}},v_{\tilde{a}}$ are enabled by the MFG-induced ME effect, $v_{\tilde{E}},$ by combining MFG-induced ME effect and shielding technique, low-frequency $v_{T}$ by the MFG-induced ME effect, and high-frequency $v_{T}$ by the low-pass FFT filter. The results in Figure 12 demonstrate a strong multisource noise suppression performance in the proposed gradient-type ME current sensor, and promising applications for small current measurement with high-level interference of ambient magnetic fields noises, electric field noises, and vibration noises.

## 5. Conclusions

We have developed a small-scale and standalone gradient-type ME current sensor for a passive and direct converting electrical current in the power cables into electrical voltages while simultaneously suppressing common-mode ambient noise from multiple sources (including magnetic fields noises, electric field noises, vibration noises, and low frequency thermal noises). The sensor is based on the product effect of the current-induced MFG effect and the MFG-induced ME effect in a magnetic biased, an electrically-shielded and mechanically-enclosed ME composite pair. The performances of the sensor porotype are systematically characterized to achieve a high sensitivity of 0.65–12.55 mV/A in the frequency range of 10 Hz–170 kHz, a strong multisource *CMRR* of 17–28 dB, a small input-output nonlinearity of <500 ppm, and a small thermal drift of <0.2%/℃ within the measurement range of 0–20 A. The high sensitivity, strong multisource *CMRR,* in conjunction with the passive, direct, and broadband current detection ability in a small-scale and standalone package, enables the use of a high reliability current measuring technique for electrical condition monitoring in hash environments such as the electrical mobility system and power plants.
